# Epidemiological characteristics and clinical predictors of neonatal jaundice in a multi- ethnic population in Xinjiang, China: a retrospective study

**DOI:** 10.3389/fpubh.2026.1785255

**Published:** 2026-05-19

**Authors:** Junyi Wang, Qin Lu, Jin Ma, Shifang Liu, Patigul Ablet, Chuanfu Qin

**Affiliations:** 1 Department of Pediatrics, The People's Hospital of Atushi, Atushi, China; 2 Department of Obstetrics and Gynecology, The People's Hospital of Atushi, Atushi, China; 3 Department of Emergency Medicine, The People's Hospital of Atushi, Atushi, China; 4 Department of Pediatrics, Maternal and Child Health Care Hospital of Kunshan, Kunshan, China

**Keywords:** multivariable logistic regression, neonatal jaundice, retrospective study, severe jaundice, Xinjiang

## Abstract

**Background:**

Neonatal jaundice is one of the most common conditions leading to neonatal hospitalization and is a preventable cause of bilirubin-induced neurological dysfunction.

**Objectives:**

This study aimed to characterize the clinical profile of neonates with jaundice admitted to the People's Hospital of Atushi City, Xinjiang and to explore the clinical predictors of severe hyperbilirubinemia.

**Methods:**

We conducted a retrospective observational study of all neonates (≤ 28 days) admitted to the neonatal unit of the People's Hospital of Atushi City with jaundice. Demographic, perinatal, and clinical variables, including ethnicity, gestational age, birth weight, age at admission, feeding type, perinatal risk factors, total serum bilirubin (TSB) at admission and discharge, phototherapy and antibiotic use, and length of stay, were extracted from electronic medical records. Severe jaundice was defined as an admission TSB at or above the 75th percentile of the cohort distribution (348.7 μmol/L). Early-onset jaundice was defined as an age at admission of ≤ 48 h. Multivariable logistic regression was applied to identify independent predictors of severe jaundice.

**Results:**

Among 261 infants (57.1% male; 90.4% born at term), the mean gestational age was 39.2 ± 1.2 weeks, and the mean birth weight was 3.35 ± 0.52 kg. Uyghur infants comprised 82.8% of the cohort, and Han infants comprised 9.6%. The mean admission TSB was 298.1 ± 79.1 μmol/L; 22.6% of infants met the definition of severe jaundice. The proportion of severe jaundice was 27.9% among Uyghur infants and 20.0% among Han infants. In adjusted analysis, early-onset jaundice was associated with lower odds of severe hyperbilirubinemia (odds ratio [OR] 0.12, 95% confidence interval [CI] 0.01–0.98), whereas pre-term birth showed a non-significant trend toward increased risk (OR 4.03, 95% CI 0.85–19.23). Ethnicity was not an independent predictor.

**Conclusion:**

Severe neonatal jaundice is common among hospitalized infants in Atushi, Xinjiang, with higher crude proportions in Uyghur infants than in Han infants. After adjustment for clinical factors, early recognition and timely admission, rather than ethnicity *per se*, appear to be the most important factors in preventing severe hyperbilirubinemia. These findings should be interpreted with caution and validated in larger multicenter cohorts.

## Introduction

Neonatal jaundice, resulting primarily from unconjugated hyperbilirubinemia, is among the most frequent conditions encountered in newborns worldwide (1–3). While most cases are benign and self-limiting, severe hyperbilirubinemia can lead to acute bilirubin encephalopathy and chronic kernicterus, which are associated with cerebral palsy, sensorineural hearing loss, gaze abnormalities, and cognitive impairment (4–7). Despite the availability of effective treatments such as phototherapy and exchange transfusion, it continues to be reported in both high- and middle-income countries and remains an important cause of preventable neurodisability (8–10).

International guidelines, including those from the American Academy of Pediatrics (AAP) and the National Institute for Health and Care Excellence (NICE), emphasize systematic risk assessment, universal or targeted bilirubin screening, use of hour-specific TSB nomograms, and early initiation of phototherapy according to gestational-age-specific thresholds (11–14). These recommendations are largely based on evidence from populations in developed countries or cities. However, their applicability to remote, multi-ethnic regions such as Xinjiang, China, may be limited by differences in genetic background, healthcare infrastructure, and cultural practices (15, 16). In this context, hospital-based data describing the burden and determinants of severe jaundice are essential for designing locally appropriate prevention and management strategies.

The primary aim of the present study was to describe the distribution of admission total serum bilirubin (TSB) and the frequency of severe jaundice among neonates in Atushi, Xinjiang, China. Secondary aims were to (1) compare bilirubin levels and the proportion of severe jaundice among Uyghur, Han, and Kyrgyz infants; (2) examine clinical correlates of admission TSB, including gestational age, feeding type, and perinatal risk factors; and (3) identify independent predictors of severe hyperbilirubinemia. By addressing these objectives, we sought to generate evidence that could inform tailored strategies for the prevention and early management of neonatal jaundice in Xinjiang and similar multi-ethnic regions.

## Methods

### Study design and setting

We performed a retrospective observational study in the neonatal unit of the People's Hospital of Atushi City, located in Kizilsu Kirghiz Autonomous Prefecture, Xinjiang, China. The hospital provides obstetric and neonatal services for both urban and surrounding rural populations and functions as a secondary referral center. The neonatal unit admits inborn and outborn infants with medical or surgical conditions, including hyperbilirubinemia.

### Study population

All neonates admitted with a clinical diagnosis of jaundice during the study period and recorded in the electronic hospital database were eligible. Inclusion criteria were: (1) postnatal age at admission ≤ 28 days; (2) documentation of jaundice with at least one bilirubin-related measurement or record during hospitalization; and (3) documented sex, gestational age, and ethnicity. For analyses involving the primary outcome and the severe-jaundice classification, a valid numeric admission TSB value was additionally required. Infants who developed jaundice after admission were included in the overall cohort but did not contribute to analyses specifically based on admission TSB. Infants with major congenital malformations incompatible with life were excluded from the present analysis. Because data were obtained retrospectively from routinely collected records and were anonymized prior to analysis, individual informed consent was waived. The study protocol was approved by the institutional ethics committee of the People's Hospital of Atushi City.

### Data collection

Clinical and demographic information was extracted from electronic medical records and nursing charts into a structured database. Variables included: demographics (sex, ethnicity, and date of birth and date of admission), perinatal characteristics (gestational age, mode of delivery, Apgar scores at 1 and 5 min, birth weight, and admission weight), feeding and onset of jaundice (primary feeding type, age at admission, and timing of jaundice onset as recorded by clinicians), perinatal and neonatal risk factors (pre-term birth, macrosomia, pneumonia, meconium-stained amniotic fluid, twin pregnancy, vomiting, birth asphyxia, maternal fever, and small-for-gestational-age status), and laboratory and treatment variables (TSB at admission and at discharge, use of phototherapy, duration of phototherapy, use of systemic antibiotics and their duration, and length of hospital stay). Ethnicity was determined from the registration information in the medical record and grouped as Uyghur, Han, Kyrgyz, Hui, or other/unknown. Gestational age categories were defined according to conventional obstetric and neonatal classification as pre-term (<37 completed weeks), term (37–41 completed weeks), and post-term (≥42 completed weeks; operationally recorded here as >41 weeks). Age at admission was calculated as the difference between the date and time of birth and the date and time of admission, expressed in days.

### Definitions of outcomes and key variables

The primary outcome of interest was admission TSB (μmol/L). For categorical analyses, severe jaundice was defined as admission TSB at or above the 75th percentile of the distribution among infants with available measurements, corresponding to 348.7 μmol/L. This threshold was selected as a pragmatic, cohort-specific marker of relatively high bilirubin burden and is close to the clinically important level of 20 mg/dL (approximately 342 μmol/L), which has been widely used in the literature to indicate severe hyperbilirubinemia or to trigger concern for bilirubin neurotoxicity risk (17–19). Early-onset jaundice was defined as admission age ≤ 48 h, and late-onset jaundice was defined as admission age >48 h.

### Statistical analysis

Continuous variables were summarized using means and standard deviations (SD). Categorical variables were expressed as counts and percentages. Among infants with valid TSB measurements, the overall distribution of admission TSB was examined, and the proportion meeting the definition of severe jaundice was calculated. Ethnic differences in mean admission TSB and in the proportion of severe jaundice were evaluated among Uyghur, Han, and Kyrgyz infants using descriptive statistics; 95% confidence intervals (CIs) for proportions were derived using Wilson's method. Multivariable logistic regression was used to identify independent predictors of severe jaundice. Candidate predictors were selected *a priori* based on clinical relevance and included sex, gestational age (continuous, in weeks), birth weight, age at admission, documented pre-term birth status, pneumonia, macrosomia, meconium-stained amniotic fluid grade III, early-onset jaundice, and ethnicity. Odds ratios (ORs) with 95% CIs and *p*-values were reported. Model fit and multicollinearity were examined using standard diagnostic procedures. Statistical significance was defined as a two-sided *p*-value of <0.05.

## Results

### Baseline characteristics

Among the 261 neonates included, 149 (57.1%) were male and 112 (42.9%) were female (Table 1). The mean gestational age was 39.18 ± 1.22 weeks, with 90.4% term infants, 4.6% pre-term infants, and 5.0% post-term infants. Mean birth weight was 3.35 ± 0.52 kg, and mean admission weight was similar at 3.34 ± 0.52 kg, while 1- and 5-min Apgar scores were high (8.90 ± 0.43 and 9.93 ± 0.27, respectively). Vaginal delivery was the most common mode of birth (56.7%; Table 1). Breastfeeding was pre-dominant, accounting for 87.0% of infants, with 12.6% receiving formula feeding and 0.4% receiving mixed feeding. Perinatal high-risk factors were relatively infrequent. Preterm birth status was documented in 4.6%, macrosomia (birth weight ≥4 kg) in 10.7%, pneumonia in 13.0%, and meconium-stained amniotic fluid grade I–III in 6.9, 5.7 and 3.4, respectively. Systemic antibiotics were used in 14.6% of infants, with a mean duration of 105.1 ± 24.9 h among those treated. Regarding ethnicity, Uyghur infants constituted the vast majority (82.8%, *n* = 216), followed by Han (9.6%, *n* = 25), Kyrgyz (6.5%, *n* = 17), other/unknown (0.8%, *n* = 2), and Hui (0.4%, *n* = 1).

**Table 1:** Descriptive statistics for neonates (≤ 28 days) admitted with jaundice.

Variable	*N* (%)	Mean ±SD
Sex		
Male	149 (57.1%)	
Female	112 (42.9%)	
Mode of delivery		
Vaginal delivery	148 (56.7%)	
Cesarean section	113 (43.3%)	
Feeding type		
Breastfeeding	227 (87.0%)	
Formula feeding	33 (12.6%)	
Mixed feeding	1 (0.4%)	
Gestational age category		
Term (37–41 weeks)	236 (90.4%)	
Post-term (>41 weeks)	13 (5.0%)	
Preterm (<37 weeks)	12 (4.6%)	
Early-onset jaundice (age at admission ≤ 48 h)		
No	207 (79.3%)	
Yes	54 (20.7%)	
Severe jaundice (admission total bilirubin ≥75th percentile)		
No	174 (66.7%)	
Yes	59 (22.6%)	
NA	28 (10.7%)	
Systemic antibiotic treatment used		
No	223 (85.4%)	
Yes	38 (14.6%)	
Perinatal risk factor: pre-term birth		
No	249 (95.4%)	
Yes	12 (4.6%)	
Perinatal risk factor: macrosomia		
No	233 (89.3%)	
Yes	28 (10.7%)	
Perinatal risk factor: pneumonia		
No	227 (87.0%)	
Yes	34 (13.0%)	
Perinatal risk factor: meconium-stained amniotic fluid I		
No	243 (93.1%)	
Yes	18 (6.9%)	
Perinatal risk factor: meconium-stained amniotic fluid II		
No	246 (94.3%)	
Yes	15 (5.7%)	
Perinatal risk factor: meconium-stained amniotic fluid III		
No	252 (96.6%)	
Yes	9 (3.4%)	
Perinatal risk factor: twin pregnancy		
No	253 (96.9%)	
Yes	8 (3.1%)	
Perinatal risk factor: vomiting		
No	259 (99.2%)	
Yes	2 (0.8%)	
Perinatal risk factor: birth asphyxia		
No	260 (99.6%)	
Yes	1 (0.4%)	
Perinatal risk factor: fever		
No	257 (98.5%)	
Yes	4 (1.5%)	
Perinatal risk factor: small for gestational age (term)		
No	260 (99.6%)	
Yes	1 (0.4%)	
Ethnicity		
Uyghur	216 (82.8%)	
Han	25 (9.6%)	
Kyrgyz	17 (6.5%)	
Other/Unknown	2 (0.8%)	
Hui	1 (0.4%)	
Gestational age (weeks)		39.18 ± 1.22
Age at admission (days)		5.10 ± 4.30
Birth weight (kg)		3.35 ± 0.52
Admission weight (kg)		3.34 ± 0.52
1-min Apgar score		8.90 ± 0.43
5-min Apgar score		9.93 ± 0.27
Admission total serum bilirubin (μmol/L)		298.11 ± 79.05
Discharge total serum bilirubin (μmol/L)		155.81 ± 40.55
Phototherapy duration (hours)		36.57 ± 14.71
Antibiotic treatment duration (hours)		105.08 ± 24.88
Length of hospital stay (days)		3.79 ± 0.98

Percentages may not sum to exactly 100.0% because of rounding.

### Admission bilirubin levels and distribution of severe jaundice

**Figure 1:**
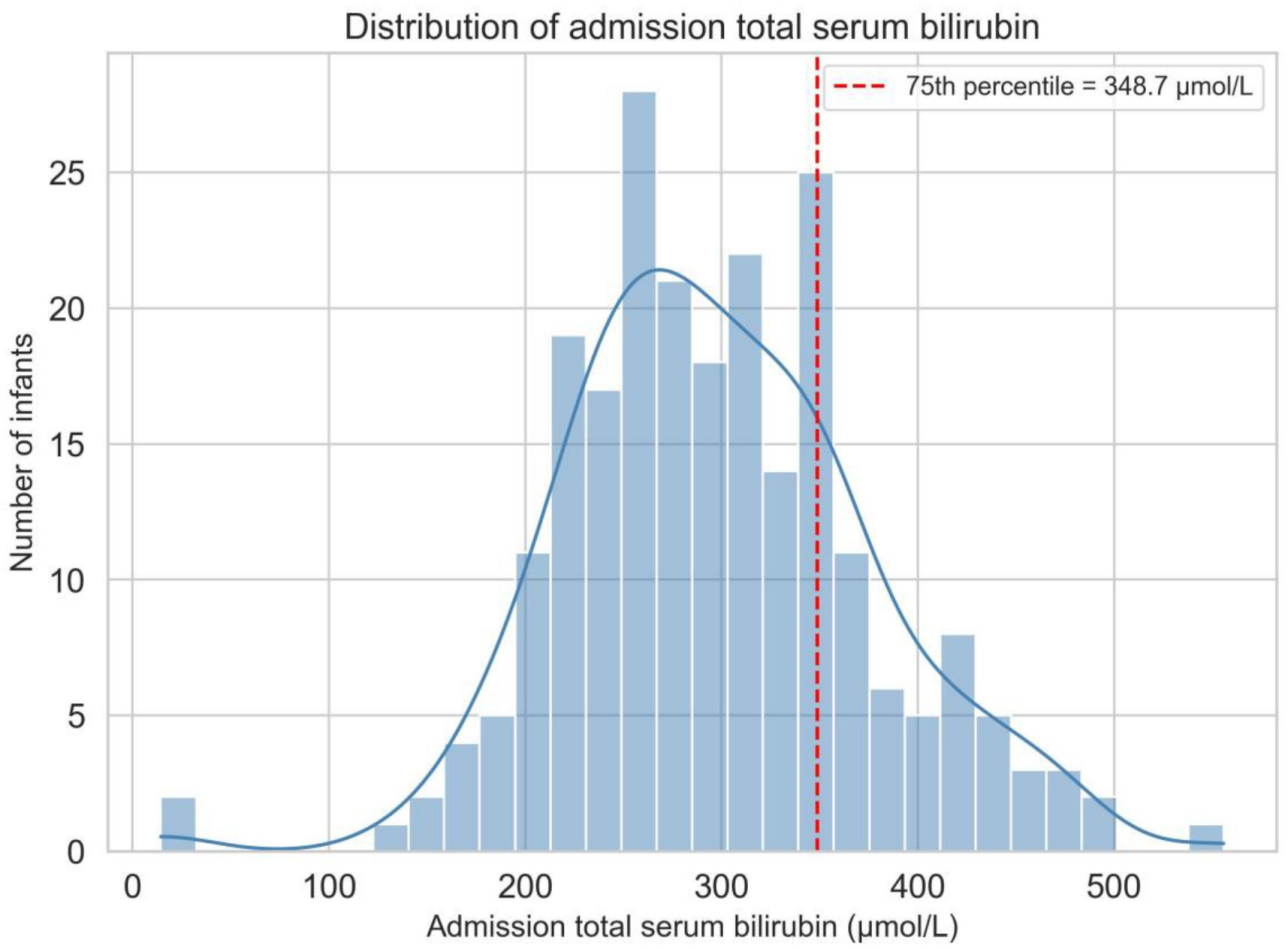
Distribution of admission total serum bilirubin among 233 neonates with available measurements. The red dashed line indicates the 75th percentile (348.7 μmol/L), used to define severe jaundice.

**Figure 2:**
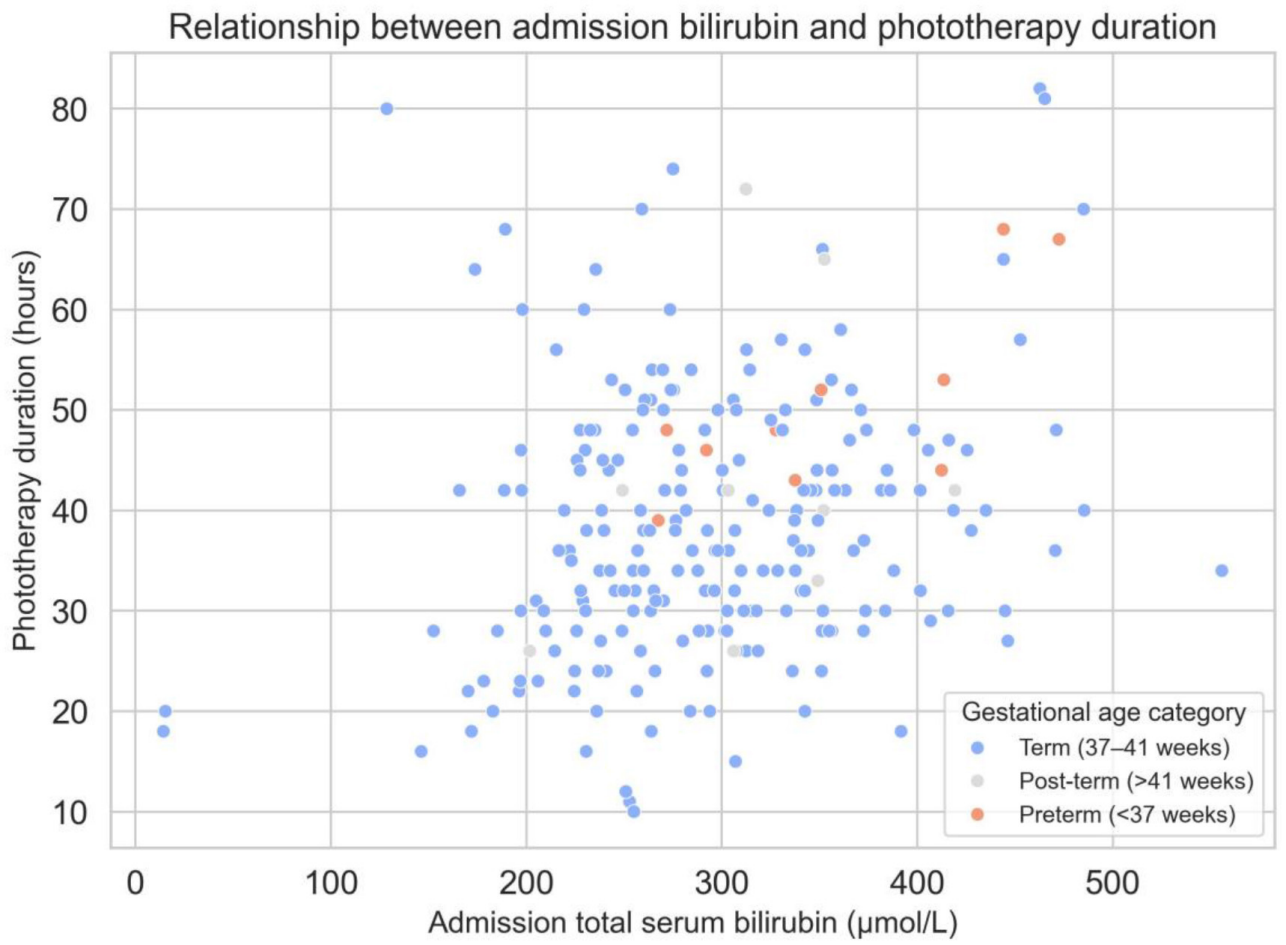
Boxplot of admission total serum bilirubin by gestational age category (pre-term, term, post-term, and missing).

Valid numeric admission TSB data were available for 233 infants. The remaining 28 infants were retained in the overall descriptive cohort because they met the general study eligibility criteria and developed jaundice during hospitalization; however, they did not have a bilirubin measurement at the time of admission and therefore could not be classified for severe jaundice, which was defined based on admission TSB values. The mean admission TSB was 298.1 ± 79.1 μmol/L (Table 1). The distribution of TSB values was approximately unimodal with slight right skewness (Figure 1). By definition, 59 infants (22.6% of the full cohort; 25.3% of those with measured TSB) had severe jaundice with admission TSB ≥348.7 μmol/L, while 174 infants (66.7%) had TSB below this threshold, and 28 were (10.7%) not classifiable due to missing admission data (Table 1). TSB at discharge was substantially lower (155.8 ± 40.6 μmol/L), consistent with the effect of treatment and the natural decline of bilirubin levels (Table 1). All infants received phototherapy during hospitalization, with a mean duration of 36.6 ± 14.7 h (Table 1). In a subset of infants, phototherapy was administered together with cefotaxime sodium antibiotic treatment according to concurrent clinical indications. Scatterplots indicated a modest positive correlation between higher admission TSB and longer phototherapy duration (Figure 2).

### Ethnic differences in bilirubin levels and severe jaundice

Among the three main ethnic groups with valid TSB data (Uyghur, Han, Kyrgyz; *n* = 230), mean admission TSB differed modestly (Table 2). Uyghur infants had the highest mean TSB (302.0 μmol/L, SD 78.9, *n* = 197), followed by Han infants (292.8 μmol/L, SD 96.3, *n* = 20) and Kyrgyz infants (253.2 μmol/L, SD 39.9, *n* = 13; Table 2). The lower variability among Kyrgyz infants likely reflects the small sample size. The proportion of severe jaundice also varied by ethnicity: 27.9% among Uyghur infants (55 of 197), 20.0% among Han infants (4 of 20), and 0% among Kyrgyz infants (0 of 13).

**Table 2:** Multivariable logistic regression for severe jaundice (odds ratios and 95% CIs).

Variable	Odds ratio	95%CI	*P*-value
Intercept	0.02	0.00–6106	0.5428
Ethnicity (Uyghur)	2.07	0.57–7.47	0.2687
Sex	1.05	0.55–1.98	0.8882
Gestational age (weeks)	1.07	0.76–1.49	0.7035
Birth weight (kg)	0.89	0.40–1.97	0.7818
Age at admission (days)	1.02	0.95–1.10	0.5440
Risk pneumonia	1.57	0.13–19.35	0.7251
Risk pre-term	4.03	0.85–19.23	0.0803
Risk macrosomia	0.99	0.28–3.53	0.9934
Risk meconium III	0.00	0.00–inf	0.9997
Early-onset jaundice	0.12	0.01–0.98	0.0474

### Multivariable predictors of severe jaundice

Regarding the onset of jaundice, 54 infants (20.7%) were admitted with early-onset jaundice (≤ 48 h) and 207 (79.3%) with later onset (Table 1). Preterm birth status showed a four-fold increase in the odds of severe jaundice (OR 4.03), but the confidence interval was wide (0.85–19.23), and the association did not reach conventional statistical significance (*p* = 0.08; Table 2). Risk factors such as pneumonia and macrosomia were not significantly associated with severe jaundice, although point estimates suggested possible modest effects. With Han infants as the reference group, the adjusted OR for severe jaundice was 2.07 (95% CI 0.57–7.47; *p* = 0.27) among Uyghur infants. Overall, after controlling for clinical variables, ethnicity *per se* was not an independent determinant of severe hyperbilirubinemia in this cohort.

## Discussion

In this retrospective study, we characterized the clinical features, ethnic distribution, and risk factors for severe neonatal jaundice among 261 hospitalized infants. Our results complement previous evidence from other regions of China and Asia. Large population-based studies have estimated that 5%−10% of term and late pre-term births develop significant hyperbilirubinemia requiring phototherapy, with higher rates in settings without universal bilirubin screening (20–22). The relatively high proportion of severe jaundice in our hospitalized cohort likely reflects a combination of referral bias and limited outpatient management capacity in a semi-rural area. Nonetheless, the mean admission TSB was approximately 300 μmol/L, and the tail of values exceeding 400–500 μmol/L underscores the ongoing risk of bilirubin neurotoxicity if diagnosis or treatment is delayed.

Ethnic differences in bilirubin metabolism and jaundice risk have been reported in the literature, with East Asian and Mediterranean populations often exhibiting higher pre-disposition due to genetic polymorphisms such as UGT1A1 variants and G6PD deficiency (23–26). In our study, Uyghur infants represented over four-fifths of all jaundiced admissions and showed higher mean TSB and a higher proportion of severe jaundice compared with Han and Kyrgyz infants. These differences might reflect a higher baseline prevalence of G6PD deficiency or hemolytic disease among Uyghur populations. However, we lacked laboratory data on G6PD enzyme status, ABO/Rh incompatibility, and direct or indirect Coombs test results, and therefore cannot confirm the underlying mechanisms. Cultural and healthcare-seeking factors may also contribute; for example, Uyghur families living in rural areas may have delayed access to hospital care, leading to higher bilirubin levels at presentation.

Interestingly, ethnicity did not emerge as a statistically significant independent predictor of severe jaundice after adjustment for gestational age, age at admission, and other variables. This may suggest that differences in timing of presentation and perinatal risk factors could account for much of the ethnic disparity in raw proportions of severe jaundice. From a practical perspective, our findings support the application of standardized risk assessment and management protocols across ethnic groups, while recognizing that targeted education and outreach in Uyghur communities might be necessary to ensure timely access to care.

Our study has several strengths. First, it provides granular clinical data on neonatal jaundice from a multi-ethnic population in western China, a region under-represented in the literature. Second, by focusing on admission TSB and using a relative threshold for severe jaundice, we captured the spectrum of hyperbilirubinemia encountered in routine practice in hospitals in Xinjiang. However, important limitations must be acknowledged. The retrospective design may have led to misclassification of risk factors such as pre-term birth, infection, or hemolysis. We lacked detailed laboratory data on blood group incompatibility, including ABO/Rh status, and on direct or indirect Coombs test results, G6PD enzyme status, sepsis-related markers, and serum albumin, all of which may influence the occurrence and severity of neonatal hyperbilirubinemia and the risk of bilirubin neurotoxicity. The absence of these variables limited our ability to evaluate hemolytic and genetic mechanisms underlying ethnic differences in bilirubin levels and may have resulted in residual confounding in the multivariable analysis. Our definition of severe jaundice was based on the 75th percentile of cohort admission TSB values rather than on postnatal-age-in-hours- and gestational-age-specific treatment thresholds recommended by current guidelines. Therefore, the severe-jaundice category in this study should be interpreted as an epidemiologic indicator of relatively high bilirubin burden within the cohort, rather than a direct surrogate for guideline-defined treatment thresholds or exchange-transfusion risk. The presence of some infants with admission TSB values below 200 μmol/L reflects the fact that cohort entry was based on a clinical diagnosis of jaundice rather than on a fixed biochemical threshold. In routine neonatal care, jaundice may prompt admission even at lower TSB levels, depending on postnatal age, gestational age, referral concerns, and the trajectory of bilirubin elevation. Another limitation is the modest sample size, particularly for Han and Kyrgyz infants and for relatively infrequent exposures such as pre-term birth and meconium-stained amniotic fluid grade III. The multivariable model was used to adjust for clinically relevant covariates selected *a priori*, but the findings require validation in larger datasets. Future studies should ideally adopt a multicenter design involving other hospitals in Xinjiang to increase sample size, improve ethnic representation, and permit more robust risk modeling.

Despite these limitations, the study provides valuable preliminary evidence to guide local quality-improvement initiatives. Standardizing bilirubin measurement for all at-risk infants, implementing universal pre-discharge screening, and ensuring structured follow-up within 48–72 h after discharge are key steps recommended by international guidelines that could be tailored to the Xinjiang context. Integration of these measures with maternal and child health programs, including education in Uyghur and Mandarin languages, may help reduce preventable cases of severe hyperbilirubinemia.

## Conclusion

In this hospital-based cohort from the People's Hospital of Atushi City in Xinjiang, China, severe neonatal jaundice was frequent among admitted infants, and Uyghur infants constituted the majority of cases. Although Uyghur infants showed higher mean bilirubin levels and a greater proportion of severe jaundice than Han and Kyrgyz infants, ethnicity was not an independent predictor after adjustment for clinical factors. These findings emphasize the importance of early identification and prompt management of neonatal jaundice in multi-ethnic settings, although confirmation in larger multicenter cohorts is needed.

## Data Availability

The original contributions presented in the study are included in the article/supplementary material, further inquiries can be directed to the corresponding author.

## References

[1] Mitra S Rennie J. Neonatal jaundice: aetiology, diagnosis and treatment. Br J Hosp Med (Lond). (2017) 78:699–704. doi: 10.12968/hmed.2017.78.12.699 29240507

[2] Diala UM Usman F Appiah D Hassan L Ogundele T Abdullahi F . Global prevalence of severe neonatal jaundice among hospital admissions: a systematic review and meta-analysis. J Clin Med. (2023) 12:3738. doi: 10.3390/jcm12113738 37297932 PMC10253859

[3] Bante A Ahmed M Degefa N Shibiru S Yihune M. Neonatal jaundice and associated factors in public hospitals of southern Ethiopia: a multi-center cross-sectional study. Heliyon. (2024) 10:e24838. doi: 10.1016/j.heliyon.2024.e24838 38312544 PMC10835243

[4] Falke M. The basics of neonatal hyperbilirubinemia. Neonatal Netw. (2025) 44:61–7. doi: 10.1891/NN-2024-0051 40068902

[5] Wickremasinghe AC Kuzniewicz MW. Neonatal hyperbilirubinemia. Pediatr Clin North Am. (2025) 72:605–22. doi: 10.1016/j.pcl.2025.04.003 40619190

[6] Chou HH Huang LC Shen SP Tsai ML Chang YC Lin HC. Neonatal jaundice is associated with increased risks of congenital anomalies of the kidney and urinary tract and concomitant urinary tract infection. Sci Rep. (2024) 14:9520. doi: 10.1038/s41598-024-59943-2 38664452 PMC11045864

[7] Chou HC Lin HC Huang KH Chang YC. Associations between neonatal jaundice and autism spectrum disorder or attention deficit hyperactivity disorder: nationwide population based cohort study. J Formos Med Assoc. (2023). 122:1150–7. doi: 10.1016/j.jfma.2023.05.010 37225632

[8] Gottimukkala SB Lobo L Gautham KS Bolisetty S Fiander M Schindler T. Intermittent phototherapy versus continuous phototherapy for neonatal jaundice. Cochrane Database Syst Rev. (2023) 3:CD008168. doi: 10.1002/14651858.CD008168.pub2 36867730 PMC9979775

[9] Par EJ Hughes CA DeRico P. Neonatal hyperbilirubinemia: evaluation and treatment. Am Fam Physician. (2023) 107:525–34. 37192079

[10] Demirel HN Ozumut SS Ovali HF. Continuous versus intermittent phototherapy in treatment of neonatal jaundice: a randomized controlled trial. Eur J Pediatr. (2024) 183:3389–96. doi: 10.1007/s00431-024-05610-7 38767694

[11] Chastain AP Geary AL Bogenschutz KM. Managing neonatal hyperbilirubinemia: an updated guideline. JAAPA. (2024) 37:19–25. doi: 10.1097/01.JAA.0000000000000120 39259272

[12] Muchowski KE. Evaluation and treatment of neonatal hyperbilirubinemia. Am Fam Physician. (2014) 89:873−8. 25077393

[13] Kemper AR Newman TB Slaughter JL Maisels MJ Watchko JF Downs SM. Clinical practice guideline revision: management of hyperbilirubinemia in the newborn infant 35 or more weeks of gestation. Pediatrics. (2022) 150:e2022058859. doi: 10.1542/peds.2022-058865 35927462

[14] American Academy of Pediatrics Subcommittee. Management of hyperbilirubinemia in the newborn infant 35 or more weeks of gestation. Pediatrics. (2004) 114:297–316. doi: 10.1542/peds.114.1.297 15231951

[15] Yu Z Han S Wu J Li M Wang H Wang J . Validation of transcutaneous bilirubin nomogram for identifying neonatal hyperbilirubinemia in healthy Chinese term and late-preterm infants: a multicenter study. J Pediatr (Rio J). (2014) 90:273–8. doi: 10.1016/j.jped.2013.08.013 24508013

[16] Yang H Li H Xia Q Dai W Li X Liu Y . UGT1A1 variants in Chinese Uighur and Han newborns and its correlation with neonatal hyperbilirubinemia. PLoS One. (2022) 17:e0279059. doi: 10.1371/journal.pone.0279059 36520959 PMC9754166

[17] Greco C Arnolda G Boo NY Iskander IF Okolo AA Rohsiswatmo R . Neonatal jaundice in low- and middle-income countries: lessons and future directions from the 2015 don ostrow trieste yellow retreat. Neonatology. (2016) 110:172–80. doi: 10.1159/000445708 27172942

[18] Zakerihamidi M Moradi A Boskabadi H. Comparison of severity and prognosis of jaundice due to Rh incompatibility and G6PD deficiency. Transfus Apher Sci. (2023) 62:103714. doi: 10.1016/j.transci.2023.103714 37164807

[19] Khurshid F Rao SP Sauve C Gupta S. Universal screening for hyperbilirubinemia in term healthy newborns at discharge: a systematic review and meta-analysis. J Glob Health. (2022) 12:12007. doi: 10.7189/jogh.12.12007 36579719 PMC9798347

[20] Dennery PA Seidman DS Stevenson DK. Neonatal hyperbilirubinemia. N Engl J Med. (2001) 344:581–90. doi: 10.1056/NEJM200102223440807 11207355

[21] Shapiro SM. Definition of the clinical spectrum of kernicterus and bilirubin-induced neurologic dysfunction (BIND). J Perinatol. (2005) 25:54–9. doi: 10.1038/sj.jp.7211157 15578034

[22] Bhutani VK Johnson LH Schwoebel A Gennaro S. A systems approach for neonatal hyperbilirubinemia in term and near-term newborns. J Obstet Gynecol Neonatal Nurs. (2006) 35:444–55. doi: 10.1111/j.1552-6909.2006.00044.x 16881988

[23] Johnson LH Bhutani VK Brown AK. System-based approach to management of neonatal jaundice and prevention of kernicterus. J Pediatr. (2002) 140:396–403. doi: 10.1067/mpd.2002.123098 12006952

[24] Watchko JF Maisels MJ. Jaundice in low birthweight infants: pathobiology and outcome. Arch Dis Child Fetal Neonatal Ed, 2003. 88(6): p. F455–8. doi: 10.1136/fn.88.6.F455 PMC1763228 14602689

[25] Wasser DE Hershkovitz I. The question of ethnic variability and the Darwinian significance of physiological neonatal jaundice in East Asian populations. Med Hypotheses. (2010) 75:187–9. doi: 10.1016/j.mehy.2010.02.017 20207085

[26] Furness A Fair F Higginbottom G Oddie S Soltani H. A review of the current policies and guidance regarding Apgar scoring and the detection of jaundice and cyanosis concerning Black, Asian and ethnic minority neonates. BMC Pediatr. (2024) 24:198. doi: 10.1186/s12887-024-04692-4 38515076 PMC10956215

